# SG-APSIC1091: Assessment of compliance to cleaning of computers by healthcare workers (HCWs) using adenosine triphosphate (ATP) measurement

**DOI:** 10.1017/ash.2023.38

**Published:** 2023-03-16

**Authors:** Kyaw Zaw Linn, Ong Chong Hui Clara, Sharifah Farhanah, Huan Xiaowei, Loo Liang Hui, Tan Hui Ru Danielle, Nur Hafizah Binte Hamed, Allie Yin Lim, Poon Chu Ying, Tang Ying Wei, Kalisvar Marimuthu

**Affiliations:** National Centre for Infectious Diseases (NCID), Singapore; Infectious Disease Research and Training Office, National Centre for Infectious Diseases (NCID), Singapore; Infectious Disease Research and Training Office, National Centre for Infectious Diseases (NCID), Singapore; Infectious Disease Research and Training Office, National Centre for Infectious Diseases (NCID), Singapore; Infectious Disease Research and Training Office, National Centre for Infectious Diseases (NCID), Singapore; National Public Health & Epidemiology Unit, National Centre for Infectious Diseases (NCID), Singapore; National Public Health & Epidemiology Unit, National Centre for Infectious Diseases (NCID), Singapore; National Public Health & Epidemiology Unit, National Centre for Infectious Diseases (NCID), Singapore; National Public Health & Epidemiology Unit, National Centre for Infectious Diseases (NCID), Singapore; National Public Health & Epidemiology Unit, National Centre for Infectious Diseases (NCID), Singapore; Infectious Disease, Tan Tock Seng Hospital, Singapore

## Abstract

**Objectives:** HCWs are recommended to wipe the computers with alcohol wipes before clinical use. Compliance assessment by direct observation is resource intensive. We used ATP measurement as a surrogate to assess the compliance to preutilization cleaning of computers. **Methods:** We conducted a pilot study to determine the median relative light unit (RLU) value reflective of preutilization cleaning of the computers. We identified values of <250, 250–500, and >500 RLU to reflect cleaned, probably cleaned, and not cleaned computers, respectively. Subsequently, we conducted a cross-sectional study of the computers in the inpatient wards in Tan Tock Seng Hospital and National Centre for Infectious Diseases. Using 3M Clean-Trace ATP swabs, we tested 5 computers in each ward: 2 computers on wheels, 2 from the nursing station, and 1 at the patients’ room entrance. All analyses were conducted using STATA version 15 software. **Results:** Between October 4 and 10, 2021, we collected 219 samples from 219 computers. Among them, 44 (20.1%) were cleaned, 49 (22.4%) were probably cleaned, and 126 (57.5%) computers were not cleaned. Higher compliance to computer cleaning was observed in COVID-19 wards [85 ATP samples; cleaned, 37 (43.5%); probably cleaned, 26 (30.6%); not cleaned, 22 (25.9%)] compared with non–COVID-19 wards [134 ATP samples; cleaned, 7 (5.2%); probably cleaned, 23 (17.2%); not cleaned, 104 (77.6%)] (*P* < .01). No significant difference was observed in compliance with cleaning computers between the ICU [30 ATP samples; cleaned, 7 (23.3%); probably cleaned, 4 (13.3%); not cleaned, 19 (63.3%)] and general wards [189 ATP samples; cleaned, 37 (19.6%); probably cleaned, 45 (23.8%); not cleaned, 107 (56.6%)] (*P* = .47). **Conclusions:** ATP swab tests can be used as a surrogate marker to assess compliance to pre-utilization cleaning of computers. Enhanced awareness of environmental hygiene may explain the higher compliance to computer cleaning observed in COVID-19 wards.

